# Construction and Validation of an Educational Technology to Promote the Health of Postmenopausal Women with Dry Eye Syndrome

**DOI:** 10.3390/ejihpe14060112

**Published:** 2024-06-12

**Authors:** Álvaro Dantas de Almeida Junior, Italla Maria Pinheiro Bezerra, Sabrina Alaide Amorim Alves, Elisa Tristan-Cheever, Thiago Salati, Luiz Carlos de Abreu

**Affiliations:** 1 Postgraduate Program in Medical Sciences, Faculdade de Medicina, Universidade de São Paulo, São Paulo 01246-903, Brazil; alvaro.dantas@usp.br (Á.D.d.A.J.); etristan-cheever@mgh.harvard.edu (E.T.-C.); thiago.salati@usp.br (T.S.); luizcarlos@usp.br (L.C.d.A.); 2 Postgraduate Program in Public Policies and Local Development, School of Sciences of Santa Casa de Misericórdia de Vitória, Vitória 29045-402, Brazil; 3 Scientific Writing Laboratoy, School of Scienses of Santa Casa de Misericórdia de Vitória, Vitória 29027-502, Brazil; sabrina1995amorim@gmail.com; 4 Postgraduate Program in Clinical Care in Nursing and Health, Universidade Estadual do Ceará, Fortaleza 60714-903, Brazil; 5 School of Medicine, University of Limerick, V94 T9PX Limerick, Ireland; 6 Postgraduate Program in Public Health and Nutrition and Health, Department of Integrated Health Education, Universidade Federal do Espirito Santo, Vitória 29075-910, Brazil

**Keywords:** educational technology, post-menopause, Dry Eye Syndrome, eye health

## Abstract

The climacteric heralds a transition from reproductive to non-reproductive life in women, often accompanied by various clinical manifestations such as dry eye, impacting their quality of life. This study focuses on systematically developing and suitability an educational digital booklet to promote eye health practices and prevent problems among postmenopausal women. The methodological approach encompassed semi-structured interviews with women diagnosed with Dry Eye Syndrome (DES), then constructing the material integrating content, script, illustrations, and layout informed by the interview findings. Subsequent validation involved assessment by 24 health experts for content, appearance, and evaluation by a target audience of 22 women. The booklet attained high suitability scores, with a Global Content Validity Index (CVI) of 0.96, indicating superior suitability as judged by experts. Additionally, it achieved a Global CVI of 0.98 for validation by the target audience. In conclusion, the educational booklet emerges as a suitable and reliable resource for promoting eye heath among DES and non-syndrome women, offering significant potential for broader application in relevant populations.

## 1. Introduction

Women undergo several phases characterized by biological and physiological changes, transitioning between reproductive and non-reproductive periods. The climacteric phase signifies this transition, marked by endocrine shifts due to declining ovarian function. This decline manifests through reduced fertility, alterations in the menstrual cycle, and the onset of menopausal symptoms, whose intensity varies across individuals and is influenced by psycho-cultural, ethnic, and social factors [1].

Menopause, defined as the permanent cessation of menstruation after 12 consecutive months of physiological amenorrhea, results from diminished ovarian follicular activity and typically commences around age 40 [2]. In developed nations, menopause commonly occurs arounds age 52, while in Brazil, it tends to fall between 48 and 50 years [3,4]. Typical menopausal onset is recognized within the age range of 40 to 54. 

The transition period is marked by somatic, physical, and emotional symptoms experienced by approximately 60% of women, significantly impacting daily activities and quality of life [5]. Short-term symptoms include vasomotor symptoms such as hot flashes, sweating, and palpitations, along with insomnia, irritability, and depression. Medium-term symptoms involve epithelial, mucous membrane, and collagen atrophy, particularly urogenital atrophy. Long-term effects encompass cardiovascular changes and alternations in bone mass. Postmenopausal late symptoms, stemming from prolonged hypoestrogenism, manifest across various organs and systems [4,6].

Eye health becomes increasingly relevant during this phase. It is often overlooked but can profoundly affect a woman’s quality of life. According to the Brazilian Ministry, the causes of visual impairment stem from a myriad of biological, social, and environmental factors, many of which can be mitigated or avoided. The World Health Organization (WHO) reports that over 80% of visual problems are preventable [7]. The loss of visual capacity precipitates adverse consequences spanning psychological effects, social status, occupational limitations, and reduced engagement in daily activities. Low visual acuity and blindness constitute significant global public health challenges, with an estimated 90% of individuals in low-income countries [5]. 

Dry Eye Syndrome (DES), known as keratoconjunctivitis sicca, affects approximately 15% of the population over 45 years old. Intriguingly, DES exhibits a higher prevalence among postmenopausal women, particularly those utilizing estrogen or estrogen-progestin combinations, though the reasons remain controversial and unexplained [8].

DES encompasses factors relating to tears in the ocular surface and is characterized by symptoms such as mild ocular discomfort, burning, foreign body sensation, photophobia, and blurred vision [4]. Moreover, it elevates the risk of corneal ulcerations and infections and compromises visual acuity [3,5]. Given the impact on eye heath on postmenopausal women, there is a pressing need for health interventions tailored to this demographic. 

Technological interventions are pivotal in imparting knowledge and fostering reflection on individual behavior, empowering individuals to take charge of their health journey. Thus, technology development emphasizing user participations holds immense value, encouraging greater engagement and adherence to the intended heath objective [9]. Educational tools like booklets are powerful for promoting health practices, particularly among postmenopausal women with Dry Eye Syndrome. They raise awareness, facilitate learning, and encourage health habits [10,11,12]. Due to lacking heath practices targeting this demographic, a digital booklet focusing on prevention and promotion could be an efficient intervention, guiding users in adopting new habits and taking charge of their self-care. Thus, this study aims to develop a suitable and educational technology, namely a digital booklet, to promote and prevent eye health problems among women in the postmenopausal period. 

## 2. Methods

**Figure 1:**
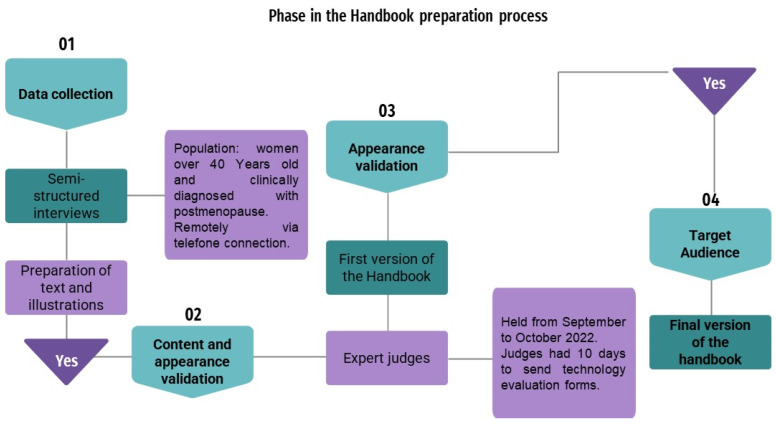
Flowchart of the methodological phase used in developing the booklet. São Paulo, SP, Brazil, 2023.

A methodological study consisting of four distinct phases (Figure 1) was undertaken. Initially, semi-structured interviews were conducted with women who had previously participated in the 2019 research project titled “Cardiac Autonomic Modulation in Post-menopausal Women with Dry Eye Syndrome”, which involved a cohort of 96 women [13]. The interviews aimed to identify the specific needs of the target audience. Data collection occurred at Ícone da Visão—Hospital dos Olhos (ICONE) in Recife, PE, Brazil, from January 2022 to August 2023. Ícone da Visão specializes in clinical care, complementary exams, and surgical treatments in ophthalmology. An analysis of the database of these women was performed within the healthcare facility’s medical records system to initiate an active search, leveraging existing contacts for outreach. Of the 49 women contacted, 20 agreed to participate in the study, based on the following inclusion criteria: woman over 40 years old and with a clinical diagnosis of postmenopause (absence of menstruation for a period longer than 12 months) or laboratory diagnosis with FSH greater than 30 UI/MI. 

The interviews were conducted remotely via telephone, with audio recording for transcription. It is important to note that complete confidentiality was maintained during the interviews, with only the voices recorded during questioning. Following the interviews, the audio recordings were transcribed and subjected to content analysis, involving several steps: pre-analysis, wherein the material was organized through initial readings to establish familiarity with the subject matter, exploration of the material to identify common characteristics or related aspects underlying content, and establishing interpretations based on the analysis [14].

In the second phase, content selection proceeded by utilizing data gathered through semi-structured interviews to craft the text for the digital booklet. This data was meticulously organized clearly and cohesively, employing accessible language to ensure comprehension by the broadest possible audience for whom the technology was intended. 

The data from the semi-structured interviews were systematically grouped, forming the foundation for developing a script to construct the digital booklet. The outlined script and accompanying illustrations were then forwarded to a professional experienced in creating visual elements and designing educational materials to prepare the digital brochure. This brochure was formatted in PDF, ensuring accessibility across various mobile and fixed devices. Moving on to the third phase, health experts judged the suitability of both content and appearance. This suitability process was conducted remotely via email/Google Docs between September and October 2022.

In determining the number of judges, Pasquali [15] suggested a range of six to 20 individuals. Recruitment of judges was facilitated through a CV platform tailored for Brazilian researchers (https://lattes.cnpq.br/ accessed on 14 July 2023), employing search terms related to eye health, educational technology, health promotion, and women’s health. 

While 88 judges were identified, 24 ultimately agreed to participate in the research. Selection criteria for these experts included meeting at least two of the following requirements: possessing specialized skills or knowledge rendering them authoritative in the field; demonstrating expertise in a specific type of study (such as Dry Eye, Health Educational Technology, Instrument Validation, Women’s Health); passing a predefined test for judge identification; or receiving a high rating from an authoritative source [16].

After selecting eligible judges, invitations were extended via email, containing a letter outlining the research objectives. Those who accepted received a kit via email, comprising two copies of the Free and Informed Consent Form (ICF), a link to access the developed electronic booklet, a questionnaire to characterize the judges, and the validation protocol. A questionnaire utilizing a Likert-type scale ranging from one to five (1—Very little; 2—Little; 3—Average; 4—A lot; and 5—Very much) was employed to assess the booklet’s content. Judges were encouraged to elaborate on their rating for items marked with options 1, 2, or 3, providing space to articulate opinions and suggestions. 

The appropriateness of figures and texts in each page were evaluated with criteria encompassing clarity of language, practical relevance, and theoretical relevance based on the experts’ judgement referencing the previous literature [17,18]. In addition, the adequacy as an educational material was assessed using a 13-itemed questionnaire having five criteria such as content, language, graphic illustration, motivation, and cultural suitability, which were extracted from the Suitability Assessment of Materials (SAM) instrument. The original SAM instrument was developed by Doak et al., which had 22 items with six domains: content, literacy demand, graphics, layout and typography, learning stimulation and motivation, and cultural appropriateness [19]. Concerning the SAM analysis, the booklet was considered as “superior” educational material if it reached between 70% and 100% of the scores; “adequate”, if between 40% and 69%; and “inadequate”, if between 0 and 39%. SAM was applied only to judges. 

The Content Validity Index (CVI) was used in the experts’ analysis of the instruments, calculated from three mathematical equations: S-CVI/Ave (mean of the content validation indexes for all the scale indexes); S-CVI/UA (proportion of items on a scale that reach scores 4 “Much” and 5 “Very much”); and the I-CVI (Content validity of individual indices). An index equal to or greater than 78% (CVI ≥ 0.78) for the individual assessment of each item was deemed acceptable [20].

A descriptive and qualitative evaluation of judges’ suggestions was conducted. The majority were accepted, thereby establishing the version for implementation with the target audience. An instrument was employed to assess the booklet’s domains of clarity and relevance to the suitability of its appearance with the target audience. This phase was conducted remotely, with a random sample of 22 women with and without DES selected based on inclusion criteria: female gender, aged 18 years or older, and possessing Internet access for questionnaire completion via the WhatsApp application. Exclusion criteria involved compromised physical or mental health hindering booklet evaluation. The Content Validity Index (CVI) was utilized to confirm the agreement on the item’s writing style (the writing must be simple, with appropriate words to capture the content), organization (the topics of the material must present a logical sequence), appearance (educational material must use illustrations, such as drawings to arouse the reader’s interest), and motivation (the message conveyed by a printed material must arouse the reader’s interest). 

The study was approved by the Research Ethics Committee of Hospital Otávia de Freitas, in accordance with Resolution No. 466/12, under opinion 2.407.176 and CAAE No. 80490717.4.0000.5200.

## 3. Results

During the preparation of the digital booklet, meticulous attention was given to ensuring its accessibility and suitability for the target audience, organizing relevant information on the subject simply and concisely. To initiate the thematic exploration, a situational diagnosis was conducted with women diagnosed with DES, aimed at identifying gaps and addressing doubts about the condition. 

Semi-structured interviews were conducted with 20 women aged 41 and 79 to gather insights. Regarding educational background, 19 participants held higher education degrees, while one had completed high school. Regarding occupation, the participants encompassed a diverse range: eight (40%) were retired, one (5%) was a housewife, two (10%) were teachers, three (15%) were civil servants, one (5%) was a doctor, three (15%) were lawyers, and two (10%) were businesswomen.

Qualitative analysis of the interview reports guided the selection of content and images for the booklet. Key areas identified included preventive measures against DES, symptoms, diagnosis, treatment, defining the pathology, eye hygiene, and addressing excessive screen usage. This data informed the development of educational technology, ensuring that all insights gleaned from the interviewees’ statements were incorporated into the material. The impetus for creating the booklet stemmed from the necessity to develop a technology that serves as a teaching resource in healthcare facilities to provide guidance and prevent DES. Utilizing the compiled data from the construction phase, a provisional storyboard (script) was formulated. The initial version of the booklet was structured into four sections: introduction, information on DES, factors and symptoms, prevention, treatment, and conclusion, Table 1. 

The content was meticulously organized to follow a logical sequence, commencing with the conceptualization of DES. It was followed by exploring its symptoms and preventive measures and concluded with guidance on treatment. Collaborating with a graphic designer, the layout and illustrations were crafted using Adobe Illustrator CS3 and Adobe InDesign CS6 for booklet layout. The researcher simulated the storyboard on a PowerPoint file, incorporating texts and images as placeholders for those featured in the booklet. These images were sourced from the internet as sketches for the professional’s depiction in the final pamphlet. 

The resulting booklet was developed in digital format, freely accessible online. It can be accessed and downloaded from any location with internet connectivity and a web browser such as Chrome, Internet Explorer, Mozilla Firefox, Safari, and Opera. The booklet measures 14.8 cm wide and 21 cm in height and is configured in landscape format. It comprises a cover, a back cover, a presentation, and the aforementioned thematic sections. The initial version, which was 19 pages long, was titled “Dry Eye Syndrome: know to control”. 

For readability, Roboto Slab font size 14 was employed for informational content. In contrast, Oswald font was utilized for the cover and subtitles, with the cover title set at sizer 40 and subtitles at size 18. Informative texts were highlighted with enlarged font and bold turquoise markers. 

The texts and narratives were crafted using short sentences and familiar language in the active voice. Additionally, actionable sentences were highlighted to propose feasible preventive measure against DES, encouraging reader engagement. The primary author, an ophthalmologist, was depicted as the character mediating the dialogue with the patient to facilitate a dialogical approach in educational material. The character’s biographical description and caricature were tailored to reflect the researchers’ practical experience and inspire educational health interactions with patients. 

The cover illustration was designed to convey the main message, “Dry Eye Syndrome: know to control”, prominently featuring the main character, the ophthalmologist, to capture the reader’s attention effectively. In this phase of the study, 24 expert judges from the health field participated, with 75% of whom were male, aged 26 to 67 years ± 9, and 75% from the Northeast region. Regarding professional training, 87.5% of the judges had experience in the area of eye heath, with an average of 2 to 40 years ± 8 in medical care and 11 years ± 8 in teaching. Notably, 91.7% had participated in events related to the topic, 79.2% had engaged in research within the field, and 75% had published scientific articles relevant to the content covered in the booklet. 

During the suitability phase encompassing appearance, content, and practical relevance, it was observed that the majority of pages in the booklet attained an Item-Level Content Validity Index (I-CVI) greater than 0.75, except page 18, which addressed theoretical relevance and obtained an I-CVI of 0.68. Across all three dimensions, the average clarity of language was 0.961, practical relevance was 0.956, and theoretical relevance was 0.961 (Table 2).

Regarding the validity of appearance measured by the SAM instrument, it was observed that 91.02% of the responses were classified as superior, showing that at this point the booklet has high standards of appearance, as illustrated in Table 3.

The first version of the booklet sent to specialists underwent adjustments recommended in the second phase of the study. These adjustments were related to: the background color of the primer, to reflect the dryness aspect; modifications of some designs to improve appearance; and replacement of terms and rewriting of the text for better understanding by the target audience, including blurred vision, burning eyes, dryness, and visual fatigue as symptoms. The recommended modifications were analyzed and considered relevant, according to the Content Validation Index and scientific literature.

Twenty-two women participated in suitability with the target audience, and they evaluated the booklet positively in terms of writing, appearance, and motivation. Regarding the organizations, it obtained a CVI above 0.75. In terms of writing style, appearance, and motivation, the items scored 1.00, demonstrating a satisfactory value for validating the technology, as shown in Table 4.

**Figure 2:**
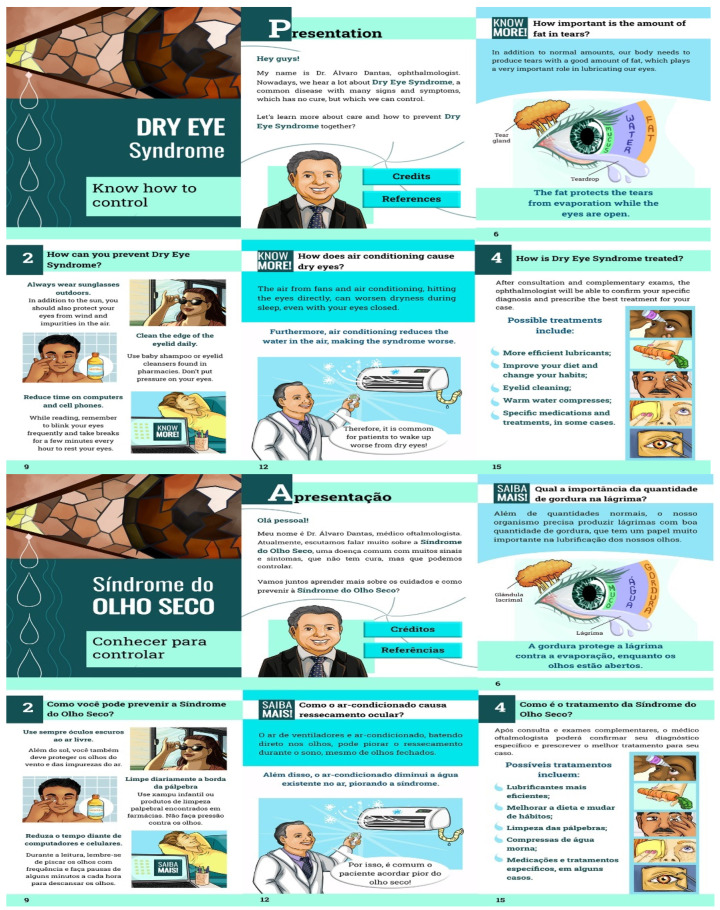
Illustration of booklet pages after suitability process with the target audience in English and in the language of the original Brazilian version. São Paulo, SP, Brazil, 2023.

Few suggestions for changes were made, all referring only to the background color of the booklet (Figure 2). Several positive comments were obtained measuring the importance of the themes included in the material, its appearance, clarity of texts, and illustrations, as expressed

*I found it important to read from beginning to end. Accessible and very didactic language. (P.02).*

*Very educational. Clear language, easy-to-read text, with brief but very important explanations. In short: clear, objective, practical, didactic and very important material (P.03).*

*I found it very interesting and educational, even as I was reading the information, I reflected on my habits and identified some points that were practical and now I will be more aware of precautions to prevent Dry Eye Syndrome (P.08).*

Therefore, it appears that this is a reliable and suitable educational material to be applied to the population in health education practices, aimed at preventing Dry Eye Syndrome and care related to eye health.

## 4. Discussion

Educational technologies, such as booklets, are efficient communication tools for health promotion. Beyond empowering users, these booklets enable individuals to act as multipliers by sharing the material with other community members [21]. Innovations in health educational technologies have transformed teaching and learning dynamics, fostering interactive relationships between professionals and users. This encourages effective learning, enhances understanding, and ensures the delivery of qualified and secure assistance [22].

Health technologies play a crucial role in driving educational practices and facilitating communication to promote self-care in health. Therefore, implementing health technologies is essential to supporting care, prevention, and health promotion initiatives for individuals [23]. It is widely recognized that the ability to engage in self-care is contingent upon one’s capacity to read and comprehend heath information. In this context, written information is a valuable complementary strategy for health education [24].

The evaluation of the booklet by expert judges revealed that it achieved acceptable values for use, with a Content Validity Index (CVI) score of 0.78 (78%). This score aligns with the parameters outlined in the scientific literature for analyzing agreement concerning health technologies. It is noteworthy that the booklet’s CVI results are consistent with those of other methodological studies that have developed and validated educational booklets [23].

It is important to highlight that the booklet was also validated by the target audience. This step is crucial because individuals may require specific visual and literary presentations depending on their educational background [25]. Feedback from the audience regarding the writing style emphasized the booklet’s relevant content and accessible language, motivating the development of eye health care practices. This underscores health technology development’s importance in addressing educational needs and facilitating communication and information access between users and health professionals [26].

Creating high-quality educational materials in the health sector enables the implementation of educational interventions grounded in structured knowledge and information for the clientele [27]. Health education is viewed as a transformative and fundamental tool in shaping comprehensive health practices. It aims to foster collaboration among various professions, specialties, services, citizens, family members, neighbors, and local social organizations to address specific health issues, strengthening and reorienting their practices, knowledge, and advocacy efforts [28].

Integrating health education practices within social movements’ pedagogical elements fosters learning for new liberating social practices [29]. Simultaneously, health promotion is recognized as a field of inquiry in public health, seeking to reorganize the care system and promote intersectoral collaboration. Embracing the socio-sanitary space concept sheds light on conflicts, resource disparities, power dynamics, and social inequalities [30].

Indeed, the empowerment of the population emerges as a novel care practice from the health promotion perspective, highlighting the effective distribution of responsibilities and rights among various stakeholders, including the State, society, individuals, and collectives [31]. Recognizing the diverse needs of social groups is integral to understanding the health–disease process, which manifest collectively and encompasses profiles of social reproduction along with their corresponding potentials and vulnerabilities. It also requires comprehension of the biological phenomena shaping the typical patterns of health and disease within these groups and individuals [32].

The notion that needs are individual and isolated is challenged as they arise within the context of transforming harmful processes stemming from relations of production and social reproduction. Consequently, needs and responses can vary depending on historical and social contexts [33,34].

From this standpoint, health education emerges as a collective health practice aimed at socializing knowledge, promoting health, and preventing diseases. It seeks to foster the development of user autonomy by incorporating practices that facilitate participatory intervention models and consider the knowledge for all involved parties. 

As a limitation of the study, it is important to note the challenges faced, such as women’s non-adherence to participation in preparing the booklet’s content and the scarcity of studies on DES and health education practices within health facilities. Disseminating the study’s results can help mitigate these gaps. 

## 5. Conclusions

The educational booklet has been deemed suitable regarding appearance and content, affirming its reliability and suitability for application among those with and without DES. Technology represents a significant asset in the realm of health, particularly for professionals providing eye health care, as educational materials serve as strategic tools for health education practices. 

## Figures and Tables

**Table 1:** Content categorization. São Paulo, Brazil, 2023.

Defined Content	Categorization
Concept of Dry Eye Syndrome	Definition of Dry Eye Syndrome, covering aspects of the disease.
Preventative care	Provides information on the main forms of prevention, essential for the reader to carry out self-care.
Symptoms	Describes the main symptoms related to Dry Eye Syndrome.
Diagnosis	Describes the main forms of examination for screening for Dry Eye Syndrome.
Excessive use of screens	It describes how excessive screen use is a mitigating factor for Dry Eye Syndrome.
Treatment	Provides essential information on forms of treatments.
Hygiene guidelines	Promote care aimed at hygiene and care if the ocular region.

**Table 2:** Distribution of Content Validity Indexes (CVI) for each item, according to analysis by expert judges. São Paulo, SP, Brazil, 2023.

Pages from the S-CVI/UA Booklet *	Assessment		
	Clarity of Language	Practical Relevance	Theoretical Relevance
P01	0.947	0.895	0.947
P02	1.000	1.000	1.000
P03	1.000	1.000	1.000
P04	1.000	1.000	1.000
P05	0.965	1.000	0.947
P06	1.000	1.000	1.000
P07	0.982	0.947	1.000
P08	1.000	1.000	1.000
P09	1.000	1.000	1.000
P10	0.825	0.895	0.842
P11	0.930	0.947	0.947
P12	1.000	1.000	1.000
P13	0.982	0.947	1.000
P14	1.000	1.000	1.000
P15	1.000	1.000	1.000
P16	1.000	1.000	1.000
P17	0.982	0.947	1.000
P18	0.737	0.737	0.684
P19	0.877	0.842	0.842
P20	0.982	0.947	1.000
P21	1.000	1.000	1.000
P22	0.895	0.895	0.895
P23	0.947	0.947	0.947
P24	1.000	1.000	1.000
S-CVI/Ave ***	0.961	0.956	0.961

* S-CVI/UA—proportion of scale items that reached a score of 4 “Much” and 5 “Very much”. *** S-CVI/Ave—average of validation indices for all scale indices.

**Table 3:** Assessment of appearance validity—SAM. São Paulo, SP, Brazil, 2023.

SAM Items	Inadequate (0)		Suitable (1)		Superior (2)	
	*n*	%	*n*	%	*n*	%
Content						
The objective is evident, facilitating the reader’s understanding.	00	0.00	00	0.00	24	100.00
The content addresses information related.	00	0.00	02	8.30	22	91.70
The proposal of the material is limited to the objectives, so that the viewer can reasonably understand it in the time allowed.	00	0.00	02	8.30	22	91.70
Language						
The reading level.	00	0.00	00	0.00	24	100.00
The conversational style makes it easier to understand the text.	00	0.00	01	4.20	23	95.80
Vocabulary uses common words.	00	0.00	05	20.80	19	79.20
Graphic illustration						
The cover attracts attention and portrays the purpose of the material.	00	0.00	07	29.20	17	70.80
The illustrations present fundamental visual messages so that the reader can understand the main points alone, without distractions.	00	0.00	02	8.30	22	91.70
Motivation						
There is interaction between the text and/or figures with the reader, leading them to solve problems, make choices, and/or demonstrate skills.	00	0.00	03	12.50	21	87.50
Desired behavior patterns are model or well-demonstrated.	00	0.00	01	4.20	23	95.80
There is motivation to develop, that is, people are motivated to learn because they believe that tasks and behaviors are feasible.	00	0.00	01	4.20	23	95.80
Suitability						
The material is culturally appropriate to the logic, language, and experience of the target audience.	00	0.00	03	12.50	21	87.50
Presents culturally appropriate images and examples.	00	0.00	01	4.20	23	95.80
Total/Average	00	0.00	2.15	8.98	21.5	91.02

**Table 4:** Distribution of Content Validity Indexes (CVI) for each item, according to analysis of the target audience. São Paulo, SP, Brazil, 2023.

Rated Items	IVC
Organization	0.983
Writing style	1.00
Appearance	1.00
Motivation	1.00
Total	0.995

## Data Availability

The data presented in this study are available on request from the corresponding author.
